# Integrated motivational interviewing and cognitive behaviour therapy can increase physical activity and improve health of adult ambulatory care patients in a regional hospital: the Healthy4U randomised controlled trial

**DOI:** 10.1186/s12889-018-6064-7

**Published:** 2018-10-11

**Authors:** Stephen Barrett, Stephen Begg, Paul O’Halloran, Michael Kingsley

**Affiliations:** 1 La Trobe University, La Trobe Rural Health School, PO Box 199, Bendigo, VIC 3552 Australia; 2 La Trobe University, School of Psychology and Public Health, Bundoora, VIC 3068 Australia

**Keywords:** Health promotion, Secondary prevention, Self-efficacy, Type 2 diabetes, Quality of life

## Abstract

**Background:**

The aim of this study was to determine whether a twelve-week, health coaching intervention could result in changes in physical activity, anthropometrics and health-related outcomes in adults presenting to an ambulatory hospital clinic.

**Methods:**

Seventy-two participants who reported being insufficiently active were recruited from an ambulatory hospital clinic and randomised to an intervention group that received an education session and eight 30-min telephone sessions of integrated motivational interviewing and cognitive behaviour therapy (MI-CBT), or to a control group that received the education session only. ActiGraph GT3X accelerometers were used to measure moderate-to-vigorous physical activity at baseline, post-intervention (3-months) and follow-up (6-months). Secondary outcome measures (anthropometrics, physical activity self-efficacy, health-related quality of life, type 2 diabetes risk) were also assessed at the three time points.

**Results:**

At baseline, the mean age and body mass index of participants (*n* = 72, 75% females) were 53 ± 8 years and 30.8 ± 4.1 kg/m^2^, respectively. Treatment group influenced the pattern of physical activity over time (*p* < 0.001). The intervention group increased moderate-to-vigorous physical activity from baseline to post-intervention and remained elevated at follow-up by 12.9 min/day (95%CI: 6.5 to 19.5 min/day). In contrast, at follow-up the control group decreased moderate-to-vigorous physical activity by 9.9 min/day (95%CI: -3.7 to -16.0 min/day). Relative to control, at follow-up the intervention group exhibited beneficial changes in body mass (*p* < 0.001), waist circumference (*p* < 0.001), body mass index (*p* < 0.001), physical activity self-efficacy (*p* < 0.001), type 2 diabetes risk (*p* < 0.001), and health-related quality of life (*p* < 0.001).

**Conclusions:**

This study demonstrates that a low contact coaching intervention results in beneficial changes in physical activity, anthropometrics and health-related outcomes that were maintained at follow-up in adults who report being insufficiently active to an ambulatory care clinic.

**Trial registration:**

ANZCTR: ACTRN12616001331426. Registered 23 September 2016,

## Background

Chronic diseases such as obesity, type 2 diabetes, and cardiovascular disease are prevalent, costly and largely preventable health conditions [1]. Almost 40% of preventable hospital admissions are due to chronic disease [2]. While the primary role of hospitals is in medical diagnosis and treatment, the increasing prevalence of chronic diseases necessitates that preventative health is included in the scope of practice for many hospital services [3]. Hospitals are important settings in which to offer health promotion interventions, particularly when delivered opportunistically alongside the provision of secondary care [3]. Hospitals provide secondary care through the delivery of non-admitted medical consultations in specialities such as general surgery, orthopaedic surgery and endocrinology. A referral from a general practitioner (GP) is required to attend a hospital specialist clinic. Patients attending secondary care hospital clinics are 40% more likely than the general population to have one or more chronic disease [2]. Therefore, hospitals offer an advantage for health promotion beyond other settings as patients experiencing ill-health are more sensitive to behaviour change contemplation, and show increased responsiveness to health advice [4]. Patients have suggested that they would like, and to an extent expect the healthcare system to provide guidance on lifestyle behaviour change and physical activity (PA) [5]. Despite the evidence-base underlining the effectiveness of health promotion services in hospitals, preventative health options for increasing self-management or lifestyle counselling around PA have been notably distant from secondary care [6].

Regular PA plays a key role in both primary prevention and management of chronic diseases [7–10]. A dose–response relationship appears to exist for PA, such that individuals with the highest levels of physical activity are at lowest risk of chronic disease [11]. Challenges remain in the translation of established research findings on the health benefits of PA into practical everyday use in the health care system [12]. Interventions that deliver prescriptive exercises can increase PA levels in hospital patients [13]; however, PA maintenance over the longer term period has proven more difficult to achieve, with more than 50% of individuals that begin an exercise program dropping out or relapsing [14]. As a result, there is an increasing use of non-traditional methods of intervention delivery to influence health behaviour change and maintenance [15].

Motivational interviewing (MI) is a behaviour change technique demonstrated to be effective in overcoming ambivalence about behaviour change [16]. MI is a person-centered, goal-orientated method of guiding participants to elicit and strengthen personal motivation and commitment to change [16]. The collaborative nature between practitioner and client contrasts MI to more prescriptive, expert-driven interventions [17]. As MI was developed to increase motivation for initial behaviour change, it has been recommended to integrate action-orientated treatments (e.g., behavioural counselling, goal-orientated therapy, cognitive behaviour therapy) to build maintenance skills [16]. A meta-analysis indicated that MI was more effective and longer-lasting when combined with another active treatment [18]. Cognitive behaviour therapy (CBT) has been increasingly integrated with MI for behaviour change [19, 20]. CBT strategies, including, but not limited to barrier identification, problem solving and self-monitoring are more goal-orientated and are used to address behaviour change across multiple health behaviour domains [19]. Integrating the theoretical underpinnings of MI and CBT together is theorised to promote long-lasting, sustained behaviour change [20]. Combined MI and CBT (MI-CBT) has resulted in small increases in PA [21–23]. Changes in PA were, however, the primary outcome in only one study [22], and all studies used self-reported outcome measures for PA change [21–23]. Self-reported measures for PA are shown to over-estimate activity when compared to objective measurement [24]. Furthermore, none of the studies recruited from outpatient secondary care clinics, where rates of chronic disease are known to be higher than the general population [25].

Hospitals are important settings from which to advocate for PA as a regular treatment for many of the lifestyle related risk factors and diseases [26]. Although doctors practicing in hospitals have stated that they do not have sufficient time to spend with patients giving advice on preventive measures [27], brief interventions in the hospital setting, such as recommendations to increase PA from clinical specialists, can have a strong effect on subsequent lifestyle choices by patients [26]. The Healthy4U intervention is an augmentation of service, where clinicians under time constraints can direct patients who might benefit from a health behaviour change intervention. This pathway offers a potential method to deliver a preventative health intervention to patients receiving secondary care, permitting hospital specialist to refer patients to a specific service tailored for them. To the best of our knowledge the Healthy4U study is the first study to examine the change and maintenance of behavioural and physiological outcomes following the integration of a behaviour change intervention into routine care for secondary care patients.

The primary aim of the Healthy4U study was to examine the effectiveness of integrated MI-CBT for change and maintenance of physical activity in insufficiently active patients presenting to an ambulatory outpatient clinic in a public hospital. Additionally, this study investigated the effectiveness of integrated MI-CBT for changes and maintenance in anthropometry, physical activity self-efficacy, type 2 diabetes risk, health related quality of life in this population.

## Methods

### Design

**Fig. 1:**
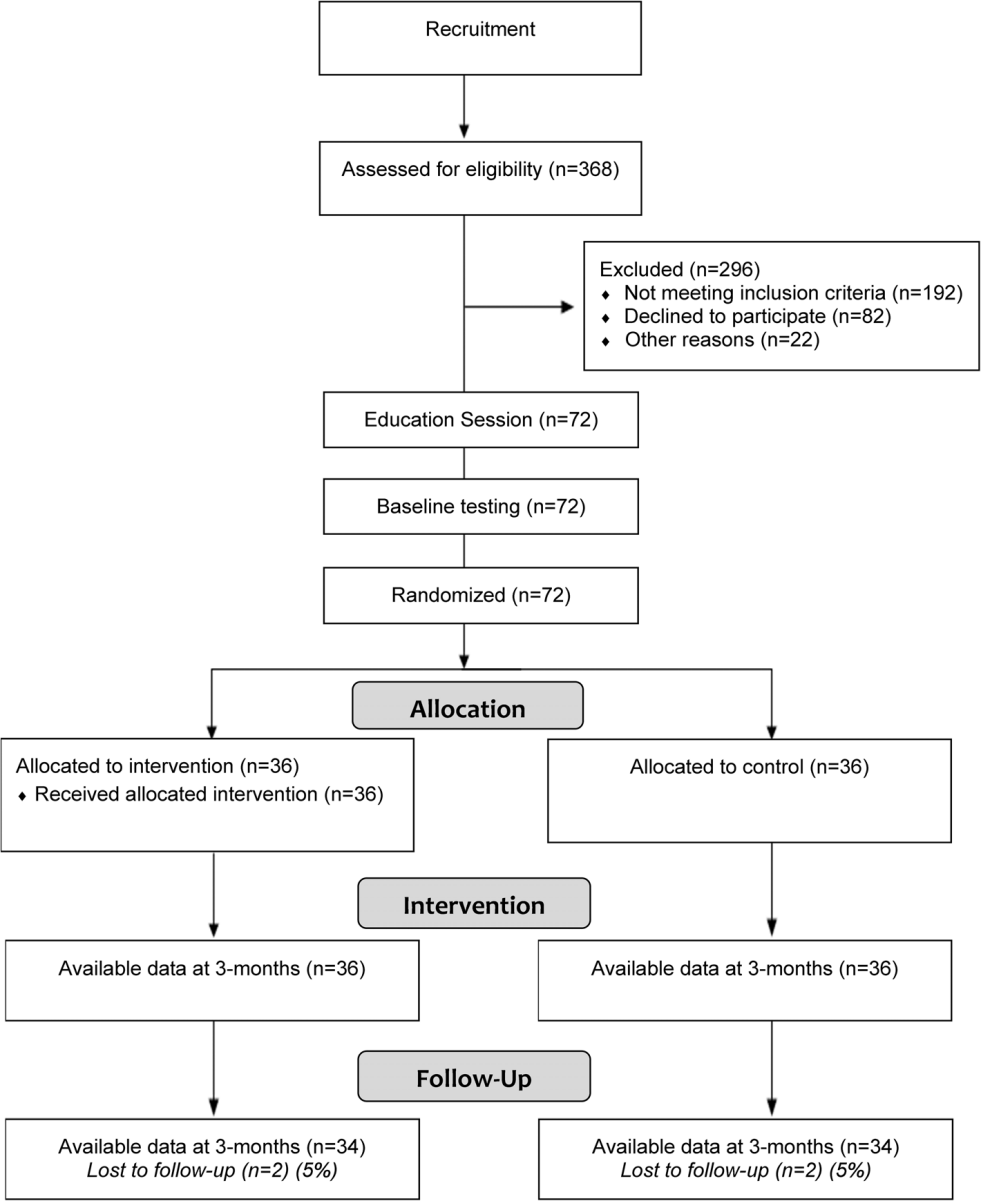
Flow of study protocol

The Healthy4U study was a single-blind randomised controlled trial designed and reported in line with the CONSORT recommendations for reporting (Fig. 1) [28]. The trial was registered with the Australian and New Zealand Clinical Trials Registry (ACTRN12616001331426) prior to patient recruitment.

### Participants

Participants were recruited from an ambulatory, secondary care clinic in a major tertiary hospital in regional Victoria. These outpatients receive medical care from specialities in general surgery, orthopaedic surgery and endocrinology after a referral from a GP. Throughout the recruitment phase of the study, recruitment flyers were available at the clinic and patients who were potentially interested in participating made direct contact with the research team using information provided on the flyer.

Participants were included if they were between 18 and 69 years, and reported being insufficiently physically active, defined as obtaining less than 150 min/week of moderate-to-vigorous physical activity (MVPA) [29]. A single item question “As a rule, do you do at least half an hour of moderate or vigorous exercise (such as walking or a sport) on five or more days of the week?” - was used to identify insufficiently physically active individuals [30]. The following exclusion criteria were applied: sufficiently physically active [29]; an existing medical condition that contraindicated PA (indicated by the Physical Activity Readiness Questionnaire); a diagnosis of diabetes; deaf/hearing impaired; disabling neurological disorder; severe mental illness such as psychosis; learning disability; dementia; registered blind; housebound or resident in nursing home; non-ambulant; pregnancy; advanced cancer.

### Randomisation and allocation

Participants who fulfilled the inclusion criteria and consented to take part in the trial were randomly allocated to either the intervention or the control group based on a random number sequence produced by a computer generated program (randomizer.org). Assignments were prepared and sealed in sequentially numbered opaque envelopes. Assignment was made by opening the next envelope in the sequence, after the recruiter had determined eligibility for the study, participants had consented to take part, attendance at an education session was confirmed and baseline measurements were completed.

### Procedure

Participants’ characteristics and outcome measures were recorded at baseline, after 3 months of intervention (post-intervention) and at 6 months (follow-up) by assessors blinded to the study group assignment. The extension of outcome measures from baseline to 6 months, which included a 3-month period where no contact with participants was made, was designed to investigate behaviour change maintenance using a previously accepted follow-up duration [31]. A recent systematic review highlighted the need for behaviour change interventions to distinguish between initial behaviour change and behaviour change maintenance [32].

### Intervention

All enrolled participants attended an education session prior to group allocation. The education session was a facilitated learning session based around self-management and lifestyle modification and was carried out using a self-determination theory (SDT) framework [33]. SDT is a general theory of human motivation that defines motivation as “psychological energy directed at a particular goal” [34]. Individuals are more likely to engage in certain behaviours, physical activity for example, if they both value that behaviour, and have motivation for change [34]. SDT was used in this group setting to support, educate and motivate participants around positive lifestyle choices [33].

The intervention group completed a telephone-based, integrated MI-CBT intervention, delivered in eight 30-min sessions over 12 weeks. The intervention was delivered using an MI framework, where MI microskills (open-ended questions, affirmations, reflections and summaries) were used in all sessions to progress participants through the MI processes of change (engagement, focusing, evocation, and planning) [16]. Throughout intervention sessions 1 to 4, MI was predominantly delivered in isolation (i.e., without CBT), exploring participants’ feelings about change and evoking intentions to change [16]. Where MI was used alone, the person delivering the intervention refrained from discussing any specific change-oriented strategies, and instead focused on exploring participant feelings and specific ambivalence regarding barriers to physical activity [16]. In the subsequent sessions, the integrated MI-CBT phase, more specific focus was directed on the identified drivers of ambivalence and resistance, leading to the formulation of goal-directed action plans [16, 35]. The CBT treatment built upon a number of evidence-based protocols with adaptations to focus on the goal of change in PA [35]. The CBT component focused more explicitly on individual determinants of PA such as PA experiences, PA outcome expectations, and PA self-efficacy [35]. The CBT strategies, which included goal setting, action planning, self-monitoring, personal feedback and relapse prevention, were incorporated within this MI framework for supporting PA change and maintenance [36]. The intervention used the integration of MI with CBT in two ways: (i) the underlying spirit of MI was used as a foundational platform from which to conduct CBT, and (ii) during more action orientated sessions therapists could switch back to MI in response to identified markers of ambivalence or resistance [35, 36].

The intervention was delivered by an experienced allied health clinician trained in MI-CBT, including workshop attendances, and one-on-one coaching from an experienced practicing psychologist. The intervener’s proficiency in using motivational interviewing was confirmed via role-play sessions, one at the commencement and one at midpoint of the intervention. Proficiency was confirmed by an independent assessor using the validated Motivational Interviewing Integrity scale 3.1.1 [37]. The intervener’s proficiency in using motivational interviewing was rated as competent on the global clinician rating at both assessments. All participants enrolled into the control arm attended the education session. Apart from contact regarding follow-up outcome measures, participants of the control group received no further contact initiated by the research team.

### Outcome measures

The primary outcome, MVPA (minutes/day) was assessed by accelerometry (wGT3X-BT; Actigraph, USA) during all waking hours over 7 consecutive days. PA was calculated using the manufacturers software (Actilife; Actigraph, USA) with cut points by Freedson Adult (1998) used to provide daily measures of MVPA (> 1951 cpm) [38]. Accelerometer wear time was based on activity counts per minute. Non-wear time was defined as 60 min or more of consecutive activity counts of zero, with a spike tolerance of 2 min and 100 cpm. Accelerometer data were considered valid if the accelerometer was worn > 10 h per day for at least 5 of 7 days including at least 1 weekend day [39]. The accelerometer was worn on a waist band, over the right hip. In adults, the hip-mounted ActiGraph has demonstrated high inter-device reliability (*r* = 0.98) and validity against indirect calorimetry (*r* = 0.56, *p* < 0.001) [40]. Participants used logbooks to report significant PA events (e.g., attending exercise class, going for a walk, heavy gardening) and periods of accelerometer non-wear. Participants returned the PA logbook along with the accelerometer within 48 h of the last accelerometer day. PA data were verified manually against the PA logbooks.

Anthropometric measures were taken objectively in accordance with International Standards for Anthropometric Assessment [41]. Waist circumference (WC) was measured to the nearest 0.1 cm using a rigid anthropometric measuring tape (Lufkin, US). Body mass was recorded to the nearest 0.1 kg using a calibrated scale (model 813; Seca, Germany). Free standing stature was recorded to the nearest 0.1 cm using a calibrated equipment with the participant barefoot (Portable stadiometer; Seca, Germany). Body mass index (BMI) was calculated by dividing body mass by the square of height. Self-efficacy to be physically active was measured using the physical activity self-efficacy survey [42]. The survey measures confidence related to undertaking physical activity over a continual timeframe with a higher score indicating a higher degree of self-efficacy. The survey has support for both its reliability and validity [42]. Health-related quality of life (HrQoL) was measured using the Medical Outcomes Study Short Form 12 Health Survey (SF-12) [43]. The SF-12 is a valid and reliable tool with published psychometric support [44]. A single index score on a scale of 0 to 1 was calculated for all participants, with a higher score indicating a more favourable health state [45].

The Australian type 2 diabetes risk assessment tool (AUSDRISK) was used to measure risk of type 2 diabetes [46]. This 12-item tool has been validated in a number of Australian studies [46]. The AUSDRISK includes questions on age, gender, waist circumference, and family history of diabetes. Potential scores range from 0 to 38 and relate to the probability of developing diabetes within the next 5 years [46]. For scores of 12–15, approximately one person in every 14 will develop diabetes [46]. For scores of 20 and above, approximately one person in every 3 will develop diabetes [46]. Demographic data were collected on participant postcode, employment status, smoking status and medical history.

### Study size

In order to detect between-group differences of 30 ± 50 (mean ± SD) minutes, the standardized mean difference, or effect size required is 0.60 [47]. A sample size of 30 participants per arm was calculated to be sufficient to detect an effect size of 0.60 or greater, with the alpha set at 0.05, and the power set at 0.80. Protecting against a drop-out rate of 20% over the 6-month study duration, 36 participants were recruited and randomised into each arm.

### Data analyses

Analyses were carried out using IBM SPSS Statistics for Windows (Version 23.0; IBM Corp., USA) and statistical significance was set at an alpha of 0.05. Data were assessed for normal distribution by Shapiro-Wilk’s [48]. Homogeneity of variances and covariances were assessed by Levene’s test and Box’s M test, respectively. Grouped data are presented as mean ± standard deviation. For the main analyses, a series of mixed-model ANOVAs (within: time; between: intervention) were used to assess the effects of the integrated MI-CBT intervention on each of the outcome variables separately. Mauchly’s test was consulted and Greenhouse–Geisser correction was applied if the assumption of sphericity was violated [48]. A significant interaction effect was interpreted to demonstrate that the change in dependent variables was influenced by intervention. Where data were in breach of Shapiro-Wilks test of normality, sensitivity analyses were performed. Data were explored for significant outliers and repeat sensitivity analyses were undertaken on data with outliers removed. Repeated sensitivity analyses provided no indication that the outliers had a significant effect on the outcome; therefore, all data were included in analyses.

A full intention-to-treat approach was used. For participants with missing data at 6-month follow-up (*n* = 2 in both groups), the last-observation-carried forward approach was adopted [49]. Repeat sensitivity analyses were undertaken on data with and without imputing the last-observation-carried forward value. The repeated sensitivity analyses provided no indication that the imputed values had a significant effect on the outcome.

## Results

A total of 72 participants (75% female) completed their baseline and 3-month assessment, and 68 participants completed the 6-month assessment (Fig. 1). Valid activity monitor data demonstrated wear time per day of 13 ± 1.5 h at baseline, 12 ± 2.2 h at 3 months and 13 ± 1.7 h at 6 months as calculated by using the manufacturer’s software (Choi algorithm) and corroborated through participant log diaries. The participants were 53 ± 8 years of age with a mean BMI of 30.8 kg/m^2^, and the majority (86%) had completed either secondary school or tertiary education. At baseline there were statistically significant differences between the groups for MVPA, where the intervention group completed lower daily MVPA than the control group, and for physical activity self-efficacy, where the control group reported higher levels of self-efficacy to be physically active (Table 1). All participants enrolled into the intervention arm received their scheduled eight sessions of integrated MI-CBT. The typical length of each session was 30 ± 3 min.

**Table 1:** Characteristics of participants at baseline

Variable	Total	Intervention	Control	*p*-value
	72	36	36	
Age (years)	53 ± 8	53 ± 8	54 ± 7	0.70 ^a^
Sex: female, n (%)	54 (75%)	28 (78%)	26 (72%)	0.58 ^a^
Stature (cm)	166 ± 8	165 ± 9	168 ± 7	0.20 ^a^
Weight (kg)	84.9 ± 9.4	84.5 ± 9.9	85.3 ± 8.9	0.72 ^a^
BMI (kg/m^2^)	30.8 ± 4.1	31.1 ± 4.0	30.5 ± 4.2	0.51 ^a^
MVPA (min/day)	31.2 ± 10.1	28.1 ± 9.9	33.3 ± 10.3	0.03 ^a^
PA Self-efficacy	31 ± 10	28 ± 8	33 ± 10	0.05 ^a^
Smoker, n (%)	23 (32%)	12 (33%)	11 (31%)	0.80 ^b^
Obesity, n (%)	38 (53%)	22 (61%)	16 (44%)	0.16 ^b^
Hypertension, n (%)	14 (20%)	9 (25%)	5 (14%)	0.23 ^b^
OA/RA, n (%)	27 (38%)	16 (44%)	11 (31%)	0.22 ^b^
Depression/anxiety, n (%)	30 (42%)	16 (44%)	14 (40%)	0.63 ^b^
Employment status, n (%)				0.43 ^b^
Full time	22 (31%)	10 (28%)	12 (33%)	
Part time	30 (42%)	18 (50%)	12 (33%)	
Unemployed	7 (10%)	4 (11%)	3 (8%)	
Retired	12 (16%)	4 (11%)	8 (22%)	
Other	1 (1%)	0	1 (4%)	
Education, n (%)				0.47 ^b^
Year 10/11	10 (14%)	4 (11%)	6 (17%)	
Year 12	22 (31%)	12 (33%)	10 (28%)	
Cert I-IV	18 (25%)	7 (20%)	11 (30%)	
Diploma	13 (18%)	9 (25%)	4 (11%)	
Bachelor or higher	9 (12%)	4 (11%)	5 (14%)	

Group data expressed as means ± standard deviations. Figures in parentheses are proportions. BMI: Body mass index; MVPA: Moderate-to-vigorous physical activity; OA: Osteoarthritis; RA: Rheumatoid arthritis. ^a^ t-test between intervention and control groups. ^b^ chi square test between intervention and control groups

**Fig. 2:**
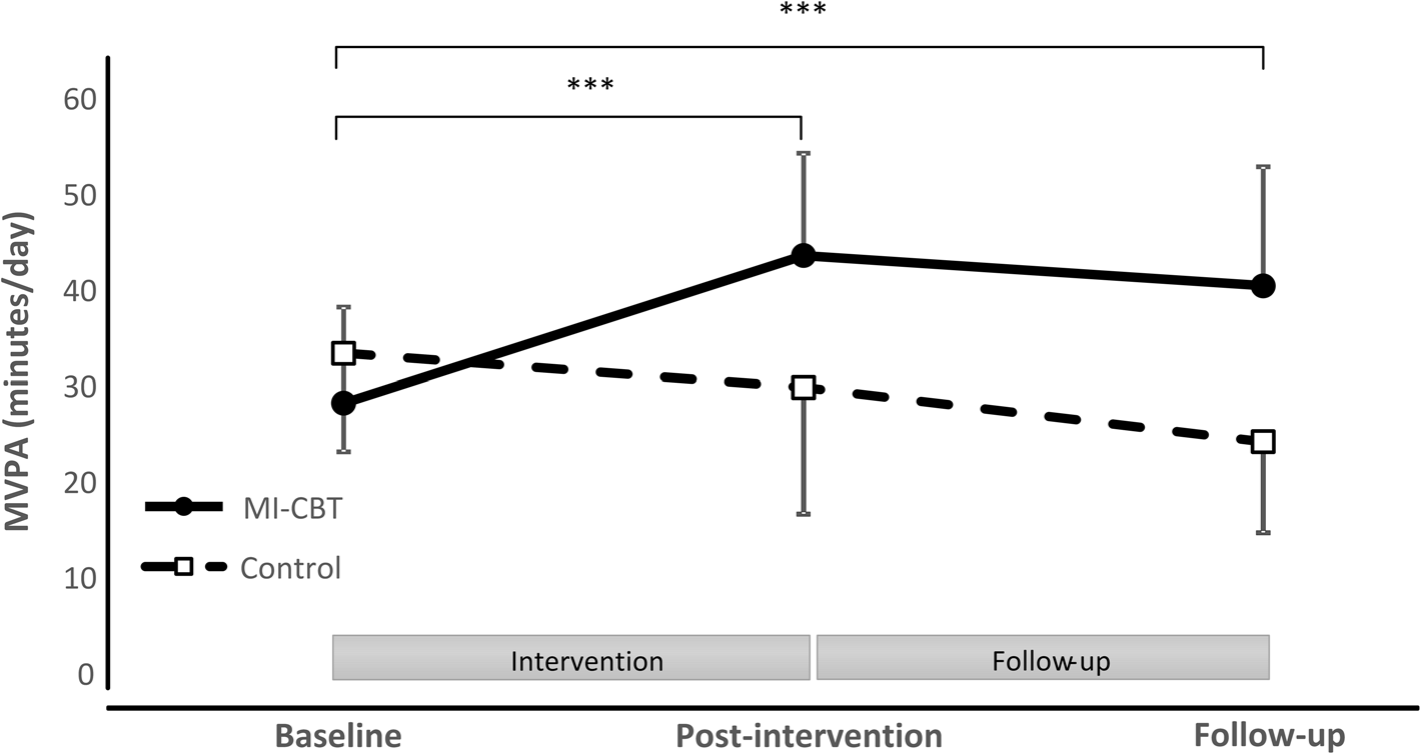
Minutes per day of moderate-to-vigorous physical activity (MVPA) for the intervention and control groups at baseline, post-intervention and follow-up. *** *p* < 0.001

There was a significant group x time interaction indicating that changes in MVPA from baseline through follow-up differed between intervention groups (*p* < 0.001; Fig. 2). The patterns for groups responded differently over time, where the intervention group significantly increased MVPA at post-intervention by 15.3 min/day (95%CI: 9.7 to 21.0 min/day), and by 12.9 min/day (95%CI: 6.5 to 19.5 min/day) at follow-up. In contrast, MVPA decreased from baseline to follow-up by 9.9 min/day (95%CI: -3.7 to -16.0 min/day) in the control group.

Statistically significant group x time interaction effects were also found for all secondary outcomes (Table 2). For the intervention group, at follow-up there were significant changes in anthropometrics, resulting in changes in WC (-2.5 cm, 95%CI: -1.8 to -3.1 cm), body mass (-2.7 kg, 95%CI: -2.1 to -3.3 kg), and BMI (-1.0 kg/m^2^, 95%CI: -0.8 to -1.2 kg/m^2^) indicating sustained changes in these variables. Relative to the control group, the intervention groups also demonstrated significant changes in physical activity self-efficacy (10 points, 95%CI: 6 to 14 points), type 2 diabetes risk (-1 risk point, 95%CI: -1 to 0 risk points) and HrQoL (0.04 units, 95%CI: 0.01 to 0.07 units).

**Table 2:** Means and standard deviations for outcome measures by time and group based on an intent-to-treat analyses

Outcome	Intervention			Control			Analyses	
	Baseline	Post-Intervention	Follow-up	Baseline	Post-Intervention	Follow-up	Time x Group (F)^a^	Effect size^b^
MVPA (min/day)	28.1 ± 9.9	43.5 ± 10.7	41.1 ± 12.5	33.3 ± 10.3	29.8 ± 13.2	23.4 ± 9.7	23.25*	0.249
Waist circumference (cm)	99.3 ± 11.7	97.2 ± 11.4	96.8 ± 11.3	96.9 ± 11.5	97.2 ± 11.4	97.3 ± 11.3	61.84*	0.469
Body mass (kg)	84.5 ± 9.9	82.5 ± 9.6	81.7 ± 9.4	85.3 ± 8.9	85.6 ± 8.8	85.7 ± 8.7	70.04*	0.500
BMI (kg/m^2^)	31.1 ± 4.0	30.4 ± 4.0	30.1 ± 3.9	30.5 ± 4.2	30.6 ± 4.1	30.7 ± 4.1	71.31*	0.505
PA self-efficacy (Risk score)	28 ± 8	36 ± 7	38 ± 7	34 ± 11	33 ± 10	32 ± 6	18.72*	0.211
HrQoL (Scale)	0.63 ± 0.08	0.62 ± 0.08	0.67 ± 0.09	0.65 ± 0.07	0.65 ± 0.08	0.62 ± 0.05	18.08*	0.205
Type 2 diabetes risk (Risk score)	14 ± 5	13 ± 4	13 ± 4	14 ± 5	14 ± 5	14 ± 5	10.91*	0.135

Group data are means ± standard deviations. *MVPA* moderate-to-vigorous physical activity, *BMI* Body mass index, *HrQoL* Health-related quality of life. *p < 0.001. ^a^ interaction effect of time by group on dependent variable; ^b^ Partial eta-squared

## Discussion

Integrated MI-CBT resulted in a meaningful increase in MVPA that was maintained at 6 months follow-up in ambulatory secondary care adults. The intervention also resulted in significant improvements in body mass, WC, BMI, PA self-efficacy, type 2 diabetes risk, and HrQoL. These improvements were maintained at 6-months, indicating a lasting effect of the intervention. This is the first study to demonstrate that an integrated MI-CBT intervention, delivered from a secondary care setting can result in significant changes in behavioural and health-related outcomes.

### Changes and maintenance in physical activity

The Healthy4U trial recruited participants deemed to be insufficiently active via self-report, and seeking to become more physically active. At baseline, daily MVPA was 31 ± 10 min, indicating moderate levels of PA. All participants completed their baseline measures after attending the education session. Interventions based on SDT have been shown to result in short-term increases in PA [50]. Therefore, it is likely that the education session contributed to moderate amounts of MVPA across all participants at baseline [50]. Despite the moderate level of PA observed at baseline, the intervention group significantly increased MVPA at 3 months (post-intervention) by a further 15.3 min/day (95%CI: 9.7 to 21.0 min/day), with similar MVPA recorded at follow-up (12.9 min/day; 95%CI: 6.5 to 19.5 min/day). These results indicate a positive change in behaviour that was maintained at 6 months. To put this change into perspective, an additional 15 min of PA is associated with a reduction in all-cause mortality of 4%, regardless of age [51]. In contrast, compared to baseline, the control group decreased MVPA at follow-up by 9.9 min/day (95%CI: -3.7 to -16.0 min/day). This reduction in MVPA in the control group is likely to reflect change from an elevated baseline value resulting from the initial education session. Decreases in PA have been shown to occur following the end of an exercise intervention, demonstrating the challenges of implementing prescriptive exercise interventions into the real world [52]. Studies have attempted to address this through the incorporation of self-monitoring strategies to prevent PA recidivism following the completion of exercise interventions [53]. A recent meta-analysis recommended that lifestyle interventions utilise self-help strategies for health related behaviour change [54]. The integration of MI and CBT provides strategies for participants to identify and overcome barriers to initiate and maintain behaviour change [20]. The observed changes in MVPA in the intervention group is suggestive of the effectiveness of integrated MI-CBT for PA change and maintenance.

### Changes and maintenance in anthropometrics

Compared to control, integrated MI-CBT resulted in significant changes in anthropometric outcomes. From baseline to follow-up, body mass reduced by 2.7 kg (95%CI: -2.1 to -3.3 kg) in the intervention group. Although the mean reduction in body mass did not exceed a clinically significant value of 5% [55], weight loss over the duration of the study is noteworthy as most middle aged individuals continue to gain weight each year [56]. The average gain in body mass of 0.5 kg (95% CI: -0.3 to 1.0) in the control exemplified the normal pattern of middle-aged weight gain [56]. The 2.5 cm decrease in WC observed in the MI-CBT group (95%CI: -1.8 to -3.1 cm) is also promising, as the baseline value of mean WC suggest an increased risk of obesity related diseases [57]. There is limited evidence for what constitutes a minimally important change in WC; however, a change of between 1.8 and 4.1 cm has been proposed as a marker of maintained change [58]. Furthermore, high waist circumference is positively associated with higher mortality rates at all levels of BMI from 20 to 50 kg/m^2^ [59]. A meta-regression analysis found that a 1 cm increase in WC can increase the relative risk of cardiovascular events by 2% [60]. The observed decrease in WC in this study is in contrast with longitudinal data on Australian adults, which reported an annualised increase in waist circumference, that did not slow over time [61]. The observed pattern of increased WC occurred with a simultaneous decrease in body mass over the period [61]. NHANES data also indicated that over time WC increased to a larger extent that expected, relative to changes in body mass over the same period [62]. These findings suggest that excess body weight over time is resulting in an increase in central adiposity, which is associated with a greater risk of cardiometabolic diseases [62]. Although recruitment into this study was based upon changing PA, not body composition, the positive changes in anthropometric measures are of clinical importance as these risk factors are strong indicators of metabolic dysfunction, and associated with development and worsening of cardiovascular disease and diabetes [63]. The changes in both WC and body mass found in this study may strengthen confidence in the MI-CBT intervention for change and maintenance in anthropometric measures.

### Changes and maintenance in health-related outcomes

The average baseline scores for PA self-efficacy indicated that the sample had moderate belief in their ability to be physically active. Lack of confidence and self-belief are strongly associated with low rates of PA [64]. Despite having had higher levels of PA self-efficacy at baseline, the mean PA self-efficacy in the control group decreased at post-intervention and even further at follow-up, which diametrically opposed the trajectory of the intervention group. This increase in PA self-efficacy is a potential mediator for the improvement and maintenance in PA levels among the intervention group. The contrast in patterns of PA self-efficacy between the groups might be explained by the exposure of the intervention group to the MI-CBT treatment. MI-CBT strategies focus on increasing self-efficacy for behaviour change, as well as developing strategies for planning and relapse prevention [16, 20]. The changes in self-efficacy found in the study participants exhibited the same pattern of change as PA. The integrated MI-CBT group increased their self-efficacy and PA, while the control group demonstrated decreases in these outcomes.

Integrated MI-CBT resulted in small but significant changes in type 2 diabetes risk. The mean baseline AUSDRISK score of 14 indicates that participants are at high risk of developing type 2 diabetes within 5 years [46]. Early screening for 2 diabetes risk, and subsequent lifestyle modification reduces the risk of type 2 diabetes and other chronic diseases [65]. Research indicates that almost 40% of individuals with prediabetes, if left untreated, will progress to diabetes in 4 years [66]. Lifestyle interventions can decrease the percentage of those with prediabetes who go on to develop diabetes to 20% [66]. The observed increase in PA and reduction in WC in the intervention group contributed to a reduction in risk score, whereas no change in risk score was observed in the control group. At follow-up the intervention group had a mean risk score of 13, which still indicates a high risk developing type 2 diabetes [46]. The long-term maintenance of behaviour change is important for the effectiveness of lifestyle interventions to prevent type 2 diabetes.

The integrated MI-CBT intervention also resulted in small but significant changes between the groups for HrQoL. A consistent positive association has been demonstrated between PA and HrQoL [67]. Changes in HrQoL in the intervention group were significant at follow-up, but not at post-intervention. Changes in multidimensional quality of life measures have been shown to be less responsive than measures of specific patient outcomes, in this example PA change, particularly where interventions are aimed at achieving a particular outcome [68]. This might account for the slower degree of change in HrQoL exhibited by the intervention group [68].

### Strengths

This study was unique in that it enrolled participants from secondary care in a public hospital, integrating preventative health into secondary care. Patients presenting to secondary care have higher rates of chronic disease than the general population, and the targeting of high risk groups is essential to address the rising prevalence of chronic diseases in Australia [69]. While hospital patients have expressed a desire for health behaviour interventions [5], research from practicing hospitals doctors indicate that they do not have sufficient time to spend giving preventive advice to patients [27]. The Healthy4U intervention was implemented to address this identified gap, to supplement clinical practice in secondary care, facilitating a process where clinicians under time duress were able to direct patients into a health behaviour intervention. With rates of lifestyle related disease projected to continue to rise in the future it is important to develop and evaluate innovative ways to address this concern [70]. For a regional hospital the delivery of the health coaching via telephone was important as it can extend reach to both geographically and socially disadvantaged areas, which commonly have higher risk of chronic diseases [71]. In RCT’s on behaviour change, the maintenance of physical activity behaviour change is not often reported, influencing the generalisability of findings [31]. The Healthy4U trial was purposely designed to assess the effect of the intervention for behaviour change and maintenance by extending outcome measures to 6 months from baseline. The use of objectively measured PA at all time points was a considerable strength of the study. Objective measures offer more precise estimates of activity intensity while removing many of the issues associated with participant recall and response bias [72]. A recent meta-analysis on MI for PA change demonstrated that the effect size of the intervention was smaller in trials using objective measures, relative to self-reported data [73]. The objectively measured changes in PA strengthen the confidence in the findings [72].

The participant retention rate in this study was high, with only 4 participants (2 from both groups) lost at follow-up. All participants were required to attend the education session, which was designed to motivate behavioural change. Mandatory attendance at the education session might have positively influenced participation in the study for individuals allocated to both groups. This study used a Manual of Operating Procedures (MOP) to facilitate consistency in protocol implementation and data collection across participants. The MOP transformed the study protocol into a guideline describing the procedure for initial and subsequent contacts with participants. The MOP included standardised procedures for reminding participants of their upcoming commitments in relation to attendance for assessments and accelerometer use, which might partially explain the strong compliance rates.

Intervention adherence rate was also high, with 100% of participants receiving all eight sessions of integrated MI-CBT. Intervention-led health behaviour change relies on mutual understanding and trust between the intervener and the participant, which can only emerge when sufficient time is given [74]. The individual delivering the intervention was confirmed as being fidelity proficient.

### Limitations

Although the target number of participants was modest, the sample size was large enough to detect significant differences between groups in all outcome measures. Recruitment of volunteers into this study meant that all participants were already interested in becoming more active. Although this might limit the transferability of findings to all community-dwelling adults, it does not influence interpretation about the effectiveness of the intervention when compared against the control, due to the robust nature of the RCT study design. As the study was confined to a 6-month timeframe, it is not clear if improvements were sustained beyond that measurement point. Nevertheless the intervention resulted in increases in MVPA that were sustained at follow-up, which is indicative of behaviour change maintenance [31], and the exhibited patterns of behaviour change found in the outcomes were not indicative of recidivism following the intervention completion [75]. Lastly, the broad generalizability of these findings might be difficult because the study was conducted in one regional location and the majority of participants were female and obese.

## Conclusion

Due to the increased prevalence of chronic disease, addressing the lifestyle behavioural mediators of these preventable diseases is essential. The Healthy4U trial demonstrates that, in comparison to control, integrated MI-CBT resulted in significant improvements in PA, anthropometrics, self-efficacy, type 2 diabetes risk and HrQoL, which were maintained at follow-up. These findings demonstrate that a behaviour change intervention implemented in secondary care is effective for the prevention and management of chronic disease.

## Abbreviations

ANOVA: Analysis of Variance
BMI: Body Mass Index
CBT: Cognitive Behaviour Therapy
CI: Confidence Interval
HrQoL: Health- Related Quality of Life
MI: Motivational Interviewing
MI-CBT: Integrated MI and CBT
MVPA: Moderate-to-Vigorous Physical Activity
NHANES: The National Health and Nutrition Examination Survey
PA: Physical Activity
RCT: Randomised Controlled Trial
SD: Standard Deviation
SDT: Self-Determination Theory
SF-12: Medical Outcomes Study Short Form 12 Health Survey
WC: Waist Circumference
